# Deciphering the potential role of post-translational modifications of histones in gastrointestinal cancers: a proteomics-based review with therapeutic challenges and opportunities

**DOI:** 10.3389/fonc.2024.1481426

**Published:** 2024-10-21

**Authors:** Reyhaneh Farrokhi Yekta, Masoumeh Farahani, Mehdi Koushki, Nasrin Amiri-Dashatan

**Affiliations:** ^1^ Proteomics Research Center, System Biology Institute, Shahid Beheshti University of Medical Sciences, Tehran, Iran; ^2^ Cancer Gene Therapy Research Center, Zanjan University of Medical Sciences, Zanjan, Iran; ^3^ Department of Clinical Biochemistry, School of Medicine, Zanjan University of Medical Sciences, Zanjan, Iran; ^4^ Zanjan Metabolic Diseases Research Center, Zanjan University of Medical Sciences, Zanjan, Iran

**Keywords:** histone, post-translational modifications, epigenetics, proteomics, gastrointestinal cancer

## Abstract

Oncogenesis is a complex and multi-step process, controlled by several factors including epigenetic modifications. It is considered that histone modifications are critical components in the regulation of gene expression, protein functions, and molecular interactions. Dysregulated post-translationally modified histones and the related enzymatic systems are key players in the control of cell proliferation and differentiation, which are associated with the onset and progression of cancers. The most of traditional investigations on cancer have focused on mutations of oncogenes and tumor suppressor genes. However, increasing evidence indicates that epigenetics, especially histone post-translational modifications (PTMs) play important roles in various cancer types. Mass spectrometry-based proteomic approaches have demonstrated tremendous potential in PTMs profiling and quantitation in different biological systems. In this paper, we have made a proteomics-based review on the role of histone modifications involved in gastrointestinal cancers (GCs) tumorigenesis processes. These alterations function not only as diagnostic or prognostic biomarkers for GCs, but a deeper comprehension of the epigenetic regulation of GCs could facilitate the treatment of this prevalent malignancy through the creation of more effective targeted therapies.

## 1 Introduction

Gastrointestinal cancers (GCs) refer to a malignant state of the gastrointestinal tract and its accessory organs, including the stomach, esophagus, liver, pancreas, small and large intestine, rectum, and anus. It accounts for about 20% of new cancer cases and 15% of cancer-associated deaths worldwide (1). Smoking, obesity, hepatitis B and C virus, and Helicobacter pylori, besides genetic mutations, are the known risk factors for GCs development, yet the specific molecular processes responsible for the initiation and advancement of these cancers are not well understood (2). In recent years, great progress has been made in understanding the role of epigenetics, including DNA methylation, histone modifications, and non-coding RNAs (such as miRNAs and lncRNAs) in carcinogenesis. It is also believed that epigenetic changes in cancer are much more common than genetic changes (3). These epigenetic modifications not only help understand tumorigenesis processes, but also can be used as potential clinical biomarkers for cancer early diagnosis, prognosis, and therapy. It is known that cancer development and metastasis are associated with epigenetic changes (4). Epigenetics refers to reversible changes in gene expression that do not affect the genome (5). Epigenetics regulates gene expression through DNA methylation, histone modifications, chromatin remodeling, and non-coding RNAs, which in turn play important roles in the activation and inactivation of oncogenes and tumor suppressors (6, 7). Post-translational modifications (PTMs) that occur in histones play critical roles in various cellular processes including transcription regulation, cell division, apoptosis, DNA damage, or DNA repair. Dysregulation of histone PTM-related pathways has been found to correlate with various human diseases such as heart failure, autoimmune diseases, and neurodegeneration (8). Additionally, the evidence also suggests a relationship between histone dysregulation and cancer (9). Modifying enzymes, including writers and erasers, trigger the induction and removal of histone modifications. Their aberrant expression is associated with disruption of the histone modification system, leading to cancer initiation, progression, and metastasis. Recently, epigenetic-based drugs have also attracted attention, many of which have been approved by the US Food and Drug Administration (FDA), but the epigenetic regulation patterns of histone modification in diseases, especially in development and progression of cancer, still need to be clarified (10). Several recent studies have scientifically evaluated the potential role of histone modifications in different types of human GCs. This review extensively examines histone post-transcriptional modifications related to GCs, primarily focusing on findings derived from proteomics methodologies.

## 2 Histone modifications

**Figure 1:**
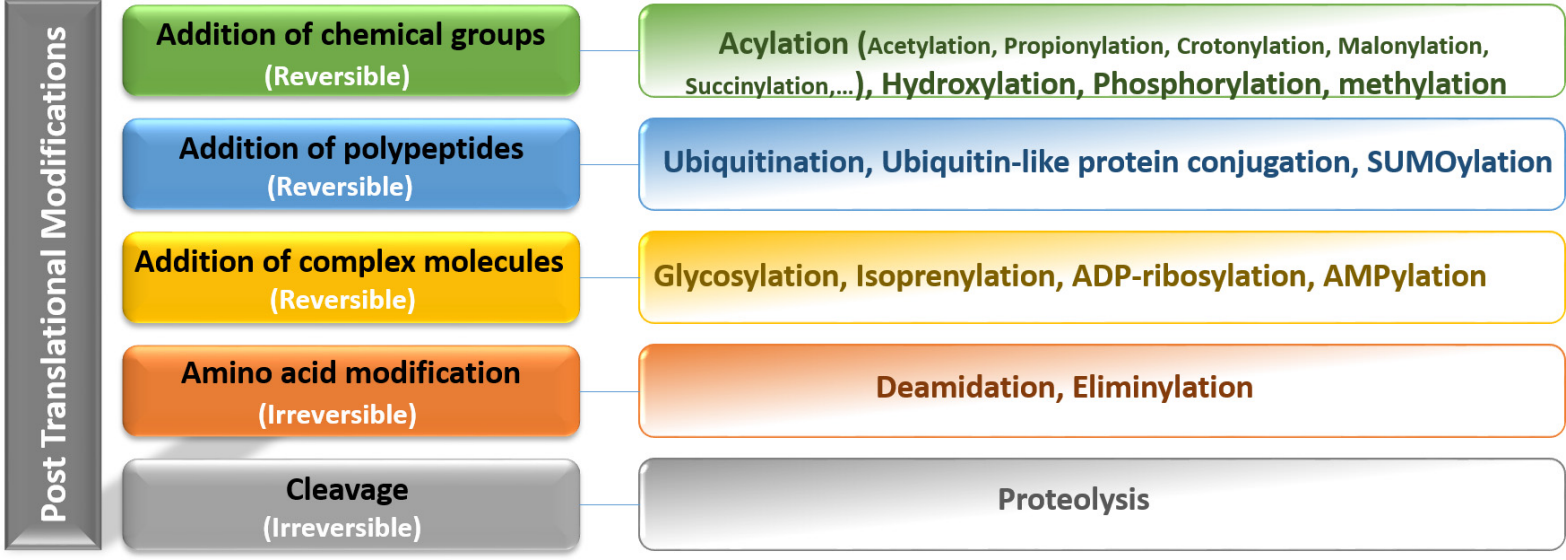
Representation of the main categories of reversible and irreversible post-translational modifications including addition of chemical groups, polypeptides, complex molecules, and amino acids. Post-translational modifications diversify the proteome by changing protein structure, location, interactions, and function and also their regulation, therefore, affecting all facets of cell biology.

Nucleosomes are the first level of DNA condensation in eukaryotes, considered the basic repeating unit in chromatin. Each nucleosome unit consists of 145 to 147 bp of DNA surrounding an octameric histone core (containing two copies of four histones: H2A, H2B, H3, and H4). Histone H1 is located in the spaces between nucleosomes along with DNA bridges (11). All histones have the similar structure, consisting of a globular domain and an unstructured N-terminal tail. Chromatin activity is regulated by the following three protein groups: i) chromatin remodeling proteins, ii) histone chaperone proteins, and iii) post-translational modification enzymes (12). Based on increasing evidence in recent years, PTMs have been found to play key roles in various cellular functions, especially in the cell cycle control, protein-protein interactions, and protein function. It has been clearly demonstrated that histone PTMs mediate a variety of important biological processes through chromatin modifications that lead to the expression or repression of target genes (13). Histone modifications are covalent additions to the N-terminal and C-terminal histone tails. Many PTMs occur in histones including acetylation, methylation, phosphorylation, ubiquitination, SUMOylation, and ADP-ribosylation of lysine (K), arginine (R), serine (S), and threonine (T) residues (14). The major PTMs are shown in **Figure 1**. Histone acetylation and methylation are the most widely investigated aberrations of histone profiles in GCs.

### 2.1 Histone acetylation

**Figure 2:**
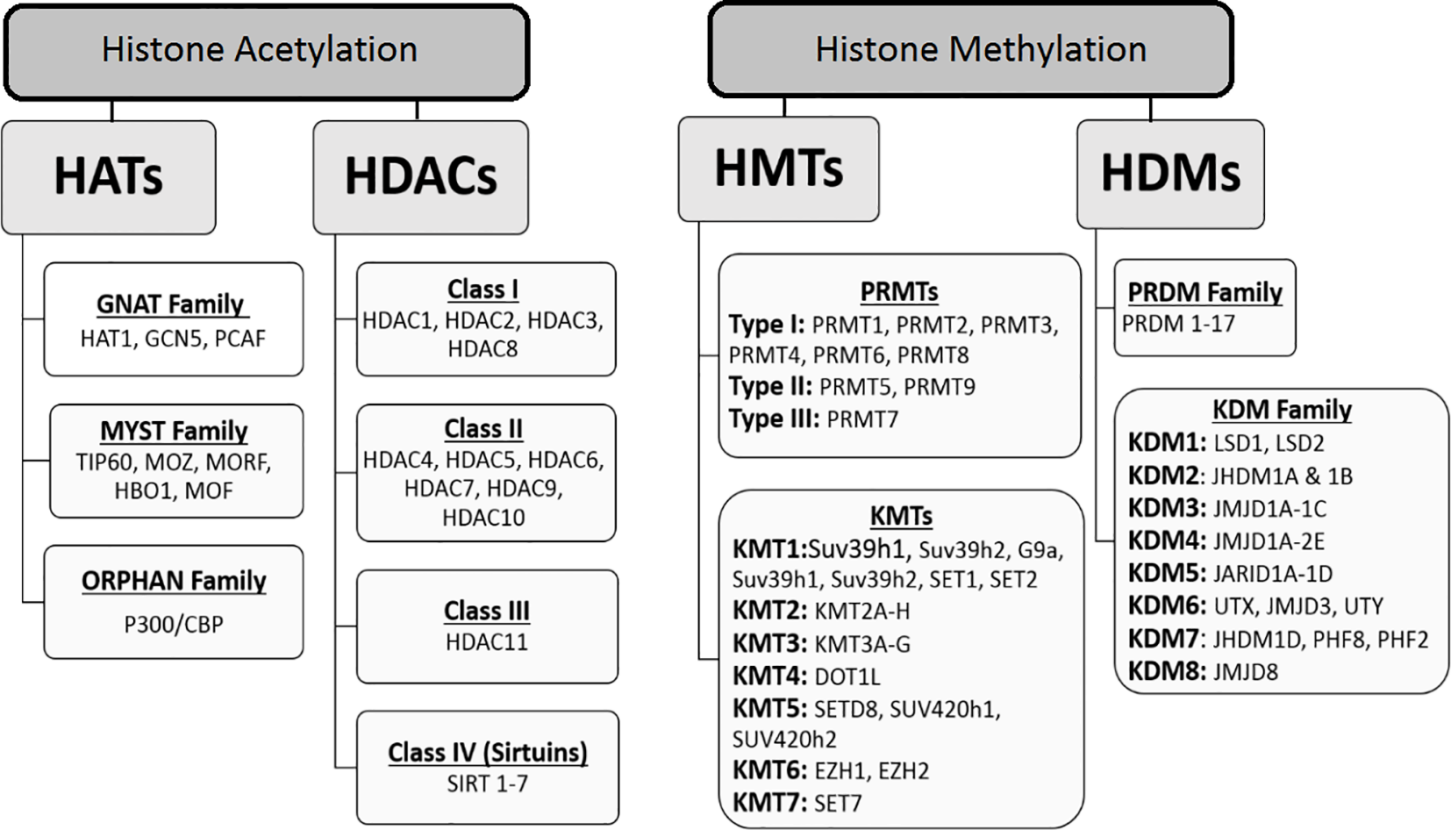
Classification of histone key modification enzymes: acetyltransferases (HATs), deacetyltransferases (HDACs), methyltransferases (HMTs) and demethyltransferases (HDMs). These enzymes catalyze the addition or removal of an array of covalent modifications in histones. In the chromatin, these modifications are involved in gene expression regulation as well as other genomic functions.

This modification is characterized by the addition of an acetyl group to certain lysine residues in the histone tail. Acetylation neutralizes the positive charge on unmodified lysine residues and modulates the interaction between negatively charged DNA and histones. These modifications influence the compaction state of chromatin and promote chromatin unfolding and transcription. Reversible acetylation of histone lysine residues is a dynamic event that is achieved by the addition or removal of histone acetyltransferases (HATs or histone acetylation “writers”) and deacetylases (HDACs or histone acetylation “erasers”) (15, 16). In addition, acetylated lysine residues serve as binding targets for “reader” proteins that recognize these modifications (17). Recently, several families of HATs have been recognized, comprising the GNAT family (including HAT1, GCN5, and PCAF), the MYST family (which consists of TIP60, MOZ, MORF, HBO1, and MOF), and the ORPHAN family (represented by P300/CBP) (18) (see **Figure 2**). The coordinated activity of these two groups of enzymes (including HAT and HDAC activity) is required for the precise regulation of gene expression (19). Several studies have shown that HATs are oncogenes and tumor suppressors, suggesting that maintaining acetylation balance is important. In addition, many HAT mutations have been identified in various human cancer (20). As a histone acetyltransferase, GCN5 is known to modulate gene transcription by facilitating the acetylation of lysine residues on several histones, including H2B, H3, and H4 (21). In addition, in the regulation of gene transcription, GCN5 can not only interact with histones but also directly acetylate transcription factors like N-Myc (22). Recent research indicates that high levels of HAT expression, such as GCN5, are frequently found in human cancers and are often associated with unfavorable clinical outcomes for cancer patients (23). Conversely, inhibiting the catalytic function of HATs through genetic or pharmacological methods can hinder the growth of cancer cells and promote their apoptosis, suggesting that HATs could serve as promising therapeutic targets for tumor chemotherapy (24). In this regard, a study conducted by Yin et al., revealed that GCN5 plays a positive role in human colon cancer development and its suppression holds a great therapeutic potential in antitumor therapy. In such a way that the referred study showed that the expression levels of GCN5 is increased in primary human colon cancers and that GCN5 suppression, by both genetic and pharmacological approaches, inhibits human colon cancer cell proliferation (25). The importance of P300/CBP has been demonstrated in many cancers, as many mutations in CBP and EP300 result in the ability of this protein to acetylate non-histone transcription factors such as p53 and BCL6 (26). In reality, the acetylation of the tumor suppressor p53 is essential for its ability to activate transcription, while, the acetylation of the proto-oncoprotein BCL6 by EP300 results in the loss of its ability to function as a transcriptional repressor. Interestingly, many mutations in the p300 leads to the loss of its acetyltransferase activity (27), indicating that the ability of p300 and CBP to acetylate proteins may have main role for their functions in growth control. Therefore, since the p300 and CBP play important roles in p53 transcriptional activity. It can be concluded that that p53 might be a crucial substrate of p300/CBP in mediating tumor suppression. According to Ito et al. study, the p300 and CBP can positively regulate p53 acetylation status (28). Overall, the tumor suppressive or oncogenic effects of HATs in cancer are dose-dependent. Overexpression is associated with oncogenic potential, while low expression results in loss of acetylation capacity. Therefore, HATs may serve as suitable drug targets. Histone acetylation erasers include four classes listed in **Figure 2**. Alterations in HDACs have been reported in cancer, but the role of each subclass of these enzymes in cancer is not yet fully understood. Mutations are rare in HDACs, but overexpression of these enzymes has been described in cancer patients (29). The activity of these enzymes and their role in cancer is not only limited to histones, as targets such as α-tubulin, HSP90, cortactin (HDAC6), p53 (HDAC5), and ERRα (HDAC8) have also been reported to be deacetylated. It has also been found that HDACs can directly affect the proteins involved in tumor growth, migration, and metastasis (13). For instance, Jung et al. demonstrated that HDAC2 directly regulates p21WAF1/CIP1 expression in a p53-independent manner and suggested that aberrant regulation of HDAC2 and its epigenetic regulation of gene transcription in apoptosis and cell cycle components play essential roles in the development of lung cancer (30). Sirtuins (SIRT 1-7) are NAD+-dependent histone deacetylases (Class III HDACs) that play a major role in the regulation of gene transcription both directly and indirectly. All sirtuins are expressed in humans, and histones H1, H3, and H4 are protein targets of histones of this class (31). Several non-histone proteins have also been reported to be deacetylated by the sirtuin family. Nonhistone sirtuin targets have been implicated in transcriptional regulation, including DNA-binding transcription factors such as forkhead box type O (FOXO) (32), p53 (33), nuclear factor-κB (NFκB) (34), and peroxisome proliferator-activated receptor-γ (PPARγ) (35). Despite evidence for a protective role of sirtuins against cancer development, some cancers are also characterized by increased levels of SIRT-1 and cancer cells depend on SIRT-1 for survival and proliferation (36, 37). In this regard, SIRT1 has been shown to silence specific tumor suppressor genes in prostate cancer cells, while it is upregulated in the nucleus and also in the cytoplasm of cancer cells (31). Among HDACs, SIRT-3 and SIRT-6 have been found to be tumor suppressor genes that regulate the glycolysis in cancer cells (38). For instance, their decreased expression is correlated with breast cancer in humans. SIRT7 is significantly overexpressed in various cancers and is associated with cancer progression (39). The “reading” of acetylated lysine in histones is performed using the bromodomain (BRD) motif in more than 40 domain-containing reader proteins. These proteins share high sequence homology, structural similarity, and play critical roles in the regulation of gene expression (40). Among these, the bromodomain and extra-terminal domain (BET) families are the most important and well-studied groups. Since acetylation is reversible, pharmacological intervention of HATs, HDACs, and lysine acetylation readers may be a potential therapeutic strategy for cancer treatment.

### 2.2 Histone methylation

Histone methylation occurs on lysine and arginine residues on histone tails. This modification is more complicated than acetylation. This modification is more complex than acetylation. There are multiple methylation states (mono, di, and trimethylation) for lysine, whereas methylation of arginine residues can be monomethylated or dimethylated (41). Histone methylation is tightly regulated by several histone methyltransferases (HMTs or “writers”) and histone demethyltransferases (HDMTs or “erasers”), whose coordinated performance is critical for gene expression, cellular and DNA stability. Some of these enzymes, such as H3K4, H3K36, and H3K79, are involved in the activation of gene transcription, while others, such as H3K9, H3K27, and H4K20, are involved in transcriptional repression (42). Lysine methylation is important in the regulation of gene expression, usually occurring on histones H3 and H4 and their sites including H3K9, H3K27, H3K36, H3K79, and H4K20 (43). Histone methylation enzymes are classified in **Figure 2**. PRMTs include Type I and Type II, which catalyze the formation of N^G^-monomethylarginine (MMA), asymmetric N^G^, N^G^-dimethylarginine (ADMA), and symmetric N^G^, N’^G^ dimethylarginine (SDMA). PRMTs are classified based on the type of methyl they accumulate: PRMT1-4 (coactivator-related arginine methyltransferase 1 [CARM1], PRMT6, and PRMT8 are Type I enzymes that produce MMA and ADMA; PRMT5 and PRMT9 are Type II enzymes that produce MMA and SDMA; and PRMT7 is a Type III enzyme that produces only MMA (44). While many PRMTs are known to methylate histone proteins, nucleosomes are methylated in some cases by PRMT5 (45) and PRMT1 (46). Recently, Fulton et al. reported that PRMT1, PRMT3, and PRMT5-8 preferentially methylate histone H4, while PRMT4 prefers histone H3 (47). Aberrant histone methylation is predominantly found in cancer. In the literature, various roles of KDM5B have been reported in cancers, and the histone demethylase KDM5B has presented itself as a therapeutic target for cancer therapy (48). As expected given the multiple roles of KDM5A and KDM5B in developmental regulation, alterations in their functions may lead to tumorigenesis. Furthermore, the regulation of angiogenesis (49), proliferation (50), motility (51), and DNA repair (52) by these two epigenetic enzymes makes them essential for cancer progression. For example, Roesch et al. demonstrated a putative tumor suppressor function for KDM5B in metastatic melanoma (53) and Mitra et al. described a proliferative role for KDM5B (54). Interestingly, Sharma et al. found that epigenetics was important in the development of drug resistance in cancer populations treated with anticancer drugs. In this regard, KDM5A has been implicated as a particular factor in the development of drug resistance. However, further research is needed in this area (55). Because dysregulated histone methylation is important in cancer development and progression, many HMDs and KDMs may be considered potential drug targets for cancer therapy. For example, studies have reported that overexpression of LSD1 occurs in a variety of cancers, including breast and prostate cancers (56). These findigs imply that LSD1 is a potential drug target for these cancers. A significant portion of small cell lung cancer (SCLC) cell lines have been shown to be remarkably sensitive to pharmacological inhibition of LSD1 (57). EZH2 has also been found to play an essential role in tumorigenesis, progression, and metastasis of cancer, and its overexpression has been observed in various types of cancer (58, 59). Furthermore, various putative drug compounds have recently been used in clinical trials against cancers such as acute myeloid leukemia (AML), non-Hodgkin lymphoma, and lung cancer, indicating the high potential of this class of compounds in cancer treatment. The discovery of histone methylation modulators has seen rapid development in recent years, but is still in its infancy. On the other hand, S-adenosylmethionine synthetase (SAMS) catalyzes the formation of S-adenosylmethionine and is essential to normal cell function. it is an important methyl donor. This means that deficiency of SAM leads to reduction in mono, di and tri histone methylation patterns in liver cancer. There are two forms of SAMs, liver-specific and nonliver-specific, which are products of two different genes. Mammalian SAMS exists as three different isoforms, designated as α (or I), β (or III), and γ (or II). The α and β forms are pertaining to the liver, whereas the γ form is widely distributed. The α and β forms are composed of four and two identical subunits and are products of the same gene. while, the γ form is a product of a separate gene and is widely distributed. It is especially highly expressed in the kidney and is often referred to as the kidney isoform. The γ form also predominates in the fetal liver and is progressively replaced by the liver-specific isoforms during development (60, 61). in this regard, several studies have shown that with the progression of hepatocarcinoma the liver-specific SAM change to kidney SAM. As stated by a few studies, the liver SAM decreases from hepatoblastoma to HCC, thus, it can be related to the lack of gene transcription of SAM mRNA. This change in gene expression may offer an advantage to cancer cells as the activity of SAMS is actually higher at physiologically relevant methionine levels. This likely represents an exploitable target for chemotherapy in a cancer lacking effective treatment currently (62).

### 2.3 Histone phosphorylation

Histone phosphorylation is another modification that occurs during DNA damage, transcriptional activation, and chromatin remodeling during cell division and apoptosis. Indeed, phosphorylation disrupts the interaction between histones and DNA that is required for the formation of homologous chromosome structures during mitosis (63). Several kinases and phosphatases regulate histone phosphorylation at PTM sites in the four histone tails, and this modification occurs primarily at serine (S), threonine (T), and tyrosine (Y) residues (64). Several kinases are involved in phosphorylation, including yTel1 and yMec1 (ATM and ATR in mammals). H3Y41 is catalyzed by the tyrosine kinase JAK2 (65). Bub1 is a kinase that catalyzes the phosphorylation of H2AT120 and H3T3 by the kinase Haspin (66). Various phosphorylation sites in histones are involved in chromatin function. For example, the combination of H3Y41 and H3K56 functions, can remarkably enhance the accessibility of DNA (67). Additionally, phosphorylation of threonine 45 in histone H3 (H3T45) is involved in apoptosis and DNA replication and may increase H3K56 acetylation (68). Histone phosphorylation is a regulatory system involved in gene expression, mainly genes associated with cell cycle regulation and proliferation. In this regard, the relationship between histone phosphorylation and cell shape changes has been demonstrated in several studies. For example, Choi et al. reported that histone H3 phosphorylation at Ser10 is an important regulatory mechanism for EGF-induced neoplastic cell transformation (69). Lau et al. also showed that histone H2B Serine 32 phosphorylation is involved in cell transformation (70). Phosphorylation of H3 at serine 10 and 28 and phosphorylation of H2B at serine 32 are involved in the activation of epidermal growth factor (EGF)-mediated gene transcription (71). In general, the complex interactions in phosphorylation can lead to gene expression and cell division. Histone H3 phosphorylation is a hallmark of mitosis mediated by Aurora kinase B. Ly-Thuy-Tram et al. suggested that Aurora kinase B is gradually activated during the entry into mitosis and anaphase onset. The complete activation of Aurora B kinase by its partners in prometaphase causes changes in the catalytic domain of Aurora B kinase that alter its affinity for ATP. These activation/inactivation cascades of Aurora B kinase correspond to different forms of the chromosome 63 complex (72). Several studies have shown that Aurora B is upregulated in many human cancers, especially in colorectal and breast cancers (73). Several Aurora kinase inhibitors have been discovered and evaluated in cancer, such as PF-03814735 and GSK1070916, which effectively block proliferation and reduce tumor growth (74). Based on the favorable results obtained with GSK1070916 in experimental and *in vitro* evaluations, this inhibitor has been advanced into the clinical trial field. PF-03814735 is a novel reversible inhibitor of the kinases Aurora1 and Aurora2 that is currently in phase I clinical trials for the treatment of advanced solid tumors. In intact cells, the inhibitory function of PF-03814735 on the kinases Aurora1 and Aurora2 decreased the levels of phospho-Aurora1, phosphohistone H3, and phospho-Aurora2. Additionally, PF-03814735 generates a block in cytokinesis, resulting in cell proliferation inhibition and the formation of polyploid multinucleated cells (75).

### 2.4 Histone ADP-ribosylation

ADP-ribosylation of histones is catalyzed by poly-ADP-ribose polymerase (PARP), with modification of this lysine residue being a relatively rare modification, occurring in 1% of histones, but particularly reported in the setting of single-strand breaks in DNA (76, 77). ADP-ribosylation of histones can be reversed by ADP-ribosylhydrolases and PAR glycohydrolases. ADP-ribosylation of histones affects DNA damage repair, replication, and transcription (78). In general, histone ribosylation induces chromatin decondensation by recruitment of DNA repair tools. Interestingly, acetylation of H4K16 inhibits ADP-ribosylation by PARP1 (79). Recent studies have also demonstrated that PARP9 plays a critical role in antiviral innate immunity by regulating target proteins through ADP-ribosylation (80, 81). Overexpression of PARP9 (BAL1), a mono-ADP-ribosyltransferase, has been reported in multiple solid tumors (82). PARP9 mediates proliferation, survival and chemo-resistance in diffuse large B-cell lymphoma (DLBCL) (83). To facilitate the manufacture of mRNA vaccine, Tan et al. identified tumor antigens and immune subtyping in colon cancer (84). In this study, PARP9 was confirmed as one of the hub genes. Patients with increased expression of these genes might be a more suitable group for mRNA vaccination. Moore et al. deciphered the role of PARP9, PARP10, and PARP14 in regulating the metabolic network and its critical cofactors (nicotinamide adenine dinucleotide (NAD) or its reduced form, NADH) in pancreatic cancer cells (85). They found that intratumoral interferon (IFN) signaling increases the consumption of NAD (H) in pancreatic cancer by up-regulation of PARP9/10/14 (85).

### 2.5 Histone ubiquitination

Histone ubiquitination is also common, usually occurring in histones H2A and H2B (86). Ubiquitination is an important modification and is quite different from other histone modifications. The ubiquitin moiety consists of a 76-amino acid polypeptide (87). It is a reversible modification that occurs in humans primarily on histone H2A at lysine 119 (H2AK119ub1) and histone H2B at lysine 120 (H2BK120ub1). Ubiquitination occurs through the formation of an isopeptide bond between the carboxy-terminal glycine of ubiquitin and the ϵ-group of a lysine residue on the carboxy-terminal tail of the histone proteins (87, 88). Histone ubiquitination occurs predominantly in the mono-ubiquitinated state that is linked to chromatin activation. It is also involved in transcriptional activation and silencing depending on the genomic context (89). H2A ubiquitination, which is associated with transcriptional suppression, may be considered a repressive factor, but H2B ubiquitination appears to be involved in both transcriptional activation and gene silencing (90, 91). The possible molecular mechanisms involved in histone ubiquitination in transcriptional regulation are limited. Several studies have reported the ability of histone H2B ubiquitination to directly promote RNA polymerase II transcription, influencing nucleosome dynamics (90). Three enzymes, including E1 activating enzyme, E2 conjugating enzyme and E3 ligase, are involved in regulating ubiquitination through sequential reactions. The E2 conjugating enzyme hHR6A/hHR6B and the E3 ligase, RNF20, are responsible for H2B ubiquitination in the cells (92, 93). The E3 ligases, Ring1B, 2A-HUB and RNF8, are responsible for H2A ubiquitination (94, 95). The deubiquitination of H2B and H2A is mediated by the DUB USP22, as part of the hSAGA complex. In addition, USP22 is required for cell cycle-related genes and also for target genes regulated by the Myc oncoprotein (96). Altered expression of enzymes involved in ubiquitination has been reported in several cancers. For example, overexpression of the E3 ligase, TRIM37, has been observed in breast cancer, promoting tumorigenesis (97). Recent studies indicate that TRIM29 plays an important role in antiviral innate immunity by regulating target proteins through protein ubiquitination (98–100) and protein SUMOylation (101). In addition, the role of TRIM29 (Tripartite Motif Containing 29) has been highlighted in several human cancers, where it can function as an oncogene, promoting tumor growth and metastasis. Xiao et al. identified TRIM29 as facilitating K48-linked ubiquitination of PHLPP1, which can activate AKT/mTOR signaling in pancreatic ductal adenocarcinoma (102). Deng et al. demonstrated that TRIM29 expression was associated with a malignant phenotype of pancreatic cancer and suggested it as a novel candidate to predict poor prognosis. They identified a novel mechanism and found TRIM29 as a regulator of YAP1 ubiquitination and its stabilizer. YAP1, as a key downstream effector in the Hippo signaling pathway, is involved in the malignant progression of tumors (103). Recent studies have reported that TRIM29 plays a significant role in colon cancer progression. Sun et al. showed upregulation of TRIM29 in colon cancer tissues and cells (104). Decreased cell viability and proliferation and KRT5 ubiquitination levels and increased protein stability and KRT5 expression were observed following the TRIM29 knockdown. Therefore, they proposed targeting TRIM29-mediated KRT5 ubiquitination levels as a potential new drug target for the treatment of colon cancer.

### 2.6 Histone SUMOylation

SUMOylation is a reversible modification that has attracted increasing attention because it mediates several biological pathways to preserve genome integrity (105). Small ubiquitin-like modifier (SUMO), which is structurally similar to ubiquitin, covalently attaches to lysine residues of specific target proteins, SUMO1, SUMO2/3 and SUMO4, which are the different detected isoforms of SUMO (106). SUMOylation plays an impressive role in regulating important biological processes such as DNA damage repair, gene expression, cell signaling, cell cycle, and apoptosis. Epithelial-mesenchymal transition (EMT) plays a key role in organogenesis and is implicated in carcinogenesis. SIRT1 plays a key role in tumorigenesis and enables ovarian cancer metastasis by preventing EMT *in vitro* and *in vivo*. The SUMO E3 ligase PIAS4 is induced by hypoxia and prevents Sp1 from binding to the SIRT1 promoter in cancer cells (107). In addition, SUMOylation plays an essential role in cell differentiation, carcinogenesis, cancer cell invasion, migration, and metastasis (108). However, the underlying molecular and cellular mechanisms remain poorly understood. Dysregulated SUMOylation leads to the development of several diseases, including cancer (109). In this regard, several human cancers have been associated with overexpression of the SUMO pathway and Sumoylation has been observed to contribute to the survival and proliferation of tumor cells as well as to the regulation of several oncogenes and tumor suppressors (109, 110). Extensive *in vitro* and *in vivo* data demonstrate the critical role of the SUMO pathway in androgen receptor (AR)-dependent signaling. Cell proliferation and hypoxia-induced angiogenesis in the prostate cancer are also regulated by the SUMO pathway through an AR-independent mechanism. Therefore, it is concluded that the SUMO pathway plays an essential role in the initiation and progression of prostate cancer, suggesting novel potential therapeutic targets (111). Chien et al. reported that the E3-type SUMO ligase PIAS4 protein (inhibitor of activated STAT protein 4) was overexpressed in pancreatic cancer cells compared with the normal pancreas (112). In another study, Bogachek et al. demonstrated that inhibition of the SUMO pathway suppressed MMP14 and CD44 expression while reducing cell invasiveness and cancer stem cell (CSC) function in colorectal cancer cell lines and primary colon cancer cells. The authors suggested that the development of SUMO inhibitors could be an advantageous strategy to target CSCs primarily in colorectal cancer, which are significantly upregulated in various cancer types and could be a potential new target for cancer therapy (113). The SUMO pathway also has an impact on protein-protein interactions and, therefore, several SUMO inhibitors have been developed (114, 115). However, a large number of human studies are needed to verify these results and provide useful information for cancer diagnosis and prognosis.

### 2.7 Histone O-GlcNAcylation

Histone O-GlcNAcylation is regulated by O-N-acetylglucosamine (O-GlcNAc) transferase (OGT) and glycoside hydrolase O-GlcNAcase (OGA), which add or remove O-GlcNAc to serine or threonine. Histone O-GlcNAcylation contributes to the mitosis cycle, chromatin dynamics, and gene expression, through crosstalk with other modifications. In several studies, a relationship between O-GlcNAc and tumorigenesis has been observed. For example, one study showed that histone deacetylase-1 (HDAC1) is over-O-GlcNAcylated in hepatocellular carcinoma (HCC) and that HCC progression could be alleviated by inhibiting O-GlcNAcylation of HDAC1 (116). In the context of breast cancer, research indicates that the expression levels of OGT are elevated in poorly differentiated tumors, and that the suppression of OGT activity can lead to a reduction in tumor growth (117).

### 2.8 Histone citrullination

Recently, histone citrullination, a PTM catalyzed by the enzyme peptidyl arginine deiminase (PAD), which has been implicated in human carcinogenesis, has been considered a novel target for tumor therapy. The PAD family enzymes consist of five isoenzymes (PAD1–4 and PAD6) with specific targets in tissues. Citrullination is dependent on high calcium concentrations (118). Under the pathological conditions, many structural proteins (such as vimentin and filaggrin) and histones, including H1, H2A, H3, and H4, are citrullinated (119, 120). PAD4 and PAD2 are overexpressed in breast cancer and deiminate R2, R8, and R17 of histone H3, ultimately leading to transcriptional repression of target genes (121). Conversely, methylation of arginine residues in histones inhibits deamination (122). Additional studies have revealed the role of histone citrullination in human diseases. Modulating histone citrullination may provide novel targets for the treatment of certain diseases (123). Therefore, PAD-mediated histone citrullination may be a promising new tumor marker with future therapeutic prospects.

### 2.9 Histone glutathionylation

This modification is a reversible modification that adds glutathione to cysteine ​​residues and contributes to DNA compaction, cell cycle progression, and DNA damage repair. Glutathione, as a physiological antioxidant, is a factor contributing to chromatin structure. Histone H3, the only histone protein containing cysteine ​​residue(s), can be modified by glutathione (GSH). The glutathionylation of histone H3 affects the stability of nucleosome structure, causing more expansion in chromatin (124). Luca et al., revealed that treatment of doxorubicin-resistant BC cells with nitric oxide resulted in histone glutathionylation, which reversed drug resistance through increased histone glutathionylation and doxorubicin storage in the nucleus (125). Therefore, glutathionylation of histone H3 opens a new window to improve the effectiveness of cancer therapies.

### 2.10 Histone lactylation

Several metabolic intermediates produced during glycolysis and mitochondrial oxidative phosphorylation play a vital role in cellular metabolism through gene regulation and histone modulation. Lactate is a main glycolysis metabolite that is produced under physiological and pathological states and plays an important role in cancer progression. Lactylation is a novel and interesting PTM with potential links to metabolic reprogramming and epigenetic remodeling and may therefore be a promising target for cancer therapy (126). The mechanism of lactation is as follows: the “writer” transfers “lactyl-CoA” to lysine residues on histones, leading to a change in its ability to bind to DNA molecules, indirectly controlling gene expression. However, the exact mechanism of histone lactation remains unclear. Zhang et al. discovered certain particular 28 lactylation sites on core histones in human and mouse cells (127). They discovered a novel epigenetic modification, lactylation, on lysine residues, that affects gene transcription from chromatin, through a mass shift from mass spectrometry that matched with a lactyl group (127). A new function for lactate via lysine lactylation (Kla) on histones was established which regulates gene expression in macrophages (128). Furthermore, the role of histone lactylation in tumorigenesis was discovered, as well as the oncogenic role found in other HMs. According to Jin et al. elevated lactate concentrations in tumor samples were associated with metastasis and poor clinical outcomes (129). The regulatory effect of lactate in the immune microenvironment is also an important tool to promote cancer progression. Recent findings suggest that lactate in the tumor microenvironment may be an immunosuppressive signal and promote tumor progression through the induction, recruitment, and regulation of immunosuppressive cells (130). In addition, lactate, by modifying histones, plays a direct role in tumor immunity by suppressing signaling pathways (131, 132). Therefore, lactate-induced lactylation designates the important role of this metabolite in tumorigenesis through epigenetic modification, as well as in a metabolic pathway mode. This point is helpful in clarifying the relationship between lactate and cancer. Interestingly, recent studies indicate the existence of crosstalk between acetylation and lactylation of histones, which may lead to the deciphering of their roles in cancer and corresponding treatment strategies. According to the survey results, histone lactylation has a slower kinetic time than histone acetylation, suggesting a higher acetylation potential than lactylation (133). Overall, lactate becomes a key point in cancer both in energy metabolism and epigenetic modification that plays a significant role in helping tumour development and progression. Thus, histone lactylation indicates a novel way for understanding the functions of lactate and its role in cancer. Furthermore, targeted lactylation may be used as an effective cancer treatment, although it is still in its infancy and requires further investigation.

## 3 Crosstalk between histone modifications

Regulation of gene transcription in an efficient manner needs cooperative function and crosstalk among several epigenetic modifications. These modifications are helpful in mediating and stabilizing signal-induced gene activation. Thus, various histone PTMs act in combination to evoke a transcriptional outcome (42). Notably, the maintenance of histone cooperation on a given gene is demonstrated via the coordinated function of epigenetic writers, readers, and erasers that create, recognize, and remove histone modifications, respectively. As noted, histones can be modified with a wide variety of PTMs, resulting in several combinatorial patterns (42). Gene transcriptional activation involves the interaction of multiple histone modifications to promote or repress gene expression. The addition or removal of a single modification can also act as a local switch to positively or negatively influence transcription (134). It is evident that some PTMs can affect the ability of other marks to be retained or read and these multiple interactions between PTMs are referred to as crosstalk. Crosstalk between histone modifications occurs when one or more histone PTMs regulate histone addition and deletion or act synergistically to promote or repress gene transcription. In addition, crosstalk can also influence the occurrence of other modifications such as DNA methylation, chromatin remodeling, and long-range chromosomal interactions (135). Some examples of crosstalk that help to understand the role of epigenetics in cancer are presented below. Lysine methylation is the most common histone modification that involves crosstalk with other histone marks, which can facilitate gene activation or repression depending on the location and state of modification. In particular, histone lysine methylation is closely linked to acetylation, where histone acetylation can promote chromatin opening and influence transcription. For example, Wang et al. demonstrated the coordinated function of H3K4me3 and histone acetylation by chromatin immunoprecipitation combined with next-generation sequencing (ChIP-seq) analysis in CD4+ T cells. The authors showed that the presence of H3K4me3 on silent genes (~30%) determines whether a gene is susceptible to histone deacetylase (HDAC) inhibition. Following HDAC inhibition, approximately two-thirds of inactive genes occupied by mono-, di-, or trimethylated H3K4 exhibited increased levels of H3K9ac and H4K16ac, and 60% of these also exhibited increased RNAPII recruitment. This evidence demonstrates the important role of H3K4 methylation as an epigenetic mark for potential gene induction through subsequent H3K9 and H4K16 acetylation. Another main crosstalk among histone modifications is histone lysine and arginine methylation. For example, the H3K4me3 appears to inhibit the deposition of the H3R2me2a mark by PRMT6. In another example, PRMT7 promotes symmetric dimethylation of H4R3 (H4R3me2s) and inhibits expression of MLL2-dependent target genes (136). Histone methylation and phosphorylation can have crosstalk in a regulatory manner. A key example is when the chromodomain recognition of a methylated lysine can be disrupted by adjacent phosphorylation. H3K9me3 is important in recruiting heterochromatin protein 1 (HP1) to distinct chromosomal domains, which is required for heterochromatin formation and gene expression regulation (137). H3Ser10 phosphorylation adjacent to the trimethylated H3 (H3K9me3S10ph) by Aurora B leads to dissociation of HP1 from heterochromatin and leads to mitotic progression (138). This event usually occurs by a disruption of the chromodomain binding of HP1 to H3K9me3 (139). Dysregulation of the described crosstalk mechanisms may lead to pathological alterations in transcriptional control, particularly in the development of cancer where several chromatin regulators have been shown to be mutational targets in various types of cancer.

## 4 Post-translational modification-specific proteomics strategies

**Figure 3:**
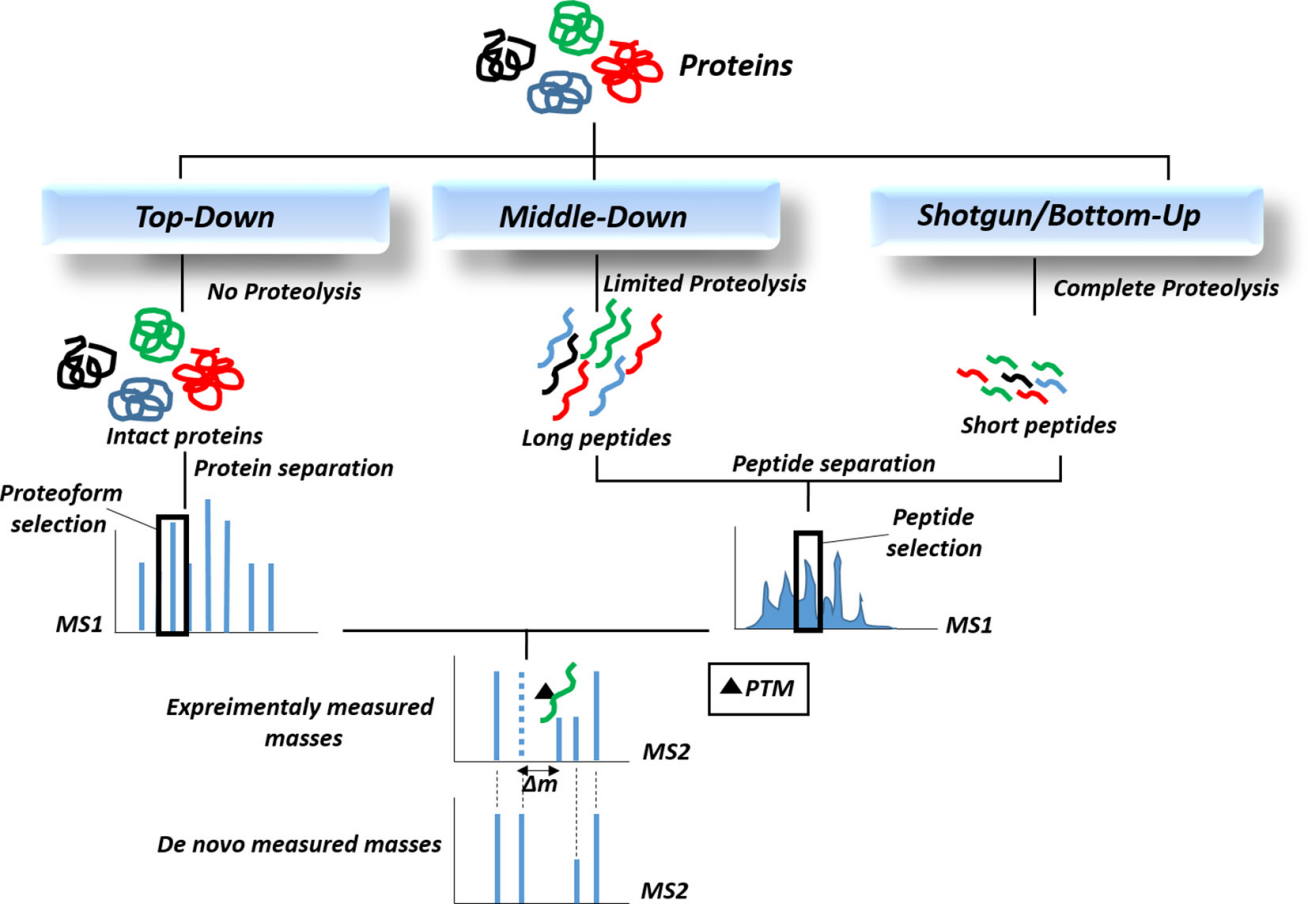
The proteomic workflows for the identification of post-translational modifications (PTMs). Since most PTMs result in a concomitant alteration in the mass of the modified protein, approaches that can identify changes in molecular mass, especially mass spectrometry-based proteomics, are now routinely utilized to detect PTMs.

Currently, mass spectrometry-based methods are the best tools for the identification and quantification of post-translational modifications in proteins and lipoproteins. The rapid development of mass-based proteomic technologies has enabled the direct identification of PTMs from complex samples such as cell lysates and tissues (140). The two main mass spectrometric methods used primarily for PTM analysis include electrospray ionization (ESI) and matrix-assisted laser desorption/ionization (MALDI) coupled with high-performance liquid chromatography. ESI mass spectrometry is particularly suitable for the analysis of PTMs in liquid samples (urine, serum, plasma, etc.). It offers several advantages for PTM analysis, including gentle ionization that preserves protein chemical structure, high ionization efficiency, and detection of PTMs in low-abundance proteins (141). MALDI mass spectrometry is also suitable for tissue biopsies and tumor samples (142). MALDI advantages include soft ionization process, high sensitivity, salt tolerance, and easy sample handling. Moreover, MALDI-imaging has been applied to identify histone PTMs in human tumor samples (142). MALDI mass imaging provides a robust tool with high resolution and high throughput for spatially resolved analysis of biomolecules directly *in situ* (143). There are 3 approaches for identification of PTMs based on mass spectrometric methods: (1) top-down approach, (2) middle-down approach, and (3) bottom-up or shotgun approach (**Figure 3**), which vary in protein digestion extent for mass analysis (144). In “Bottom-Up” proteomics, peptides are generated from enzymatic proteolysis of proteins prior to analysis in a mass spectrometer. To reduce sample complexity, fractionation techniques are used such as multidimensional protein identification technology approach (MudPIT), where peptides are further separated by strong cation exchange or reversed-phase chromatography prior to mass spectrometry. Top-down proteomics yields 100% sequence coverage where PTM combinations are also preserved for precise identification of proteins (145). “Middle-down” has emerged as a novel hybrid approach with high sequence coverage that compensates for the low throughput issue of top-down method (144). The separated proteins and peptides are then further fragmented by second mass spectrometry (MS2) to identify PTMs based on their mass shifts (Δm). Due to the low abundance of modified proteins in biological samples, efficient strategies are required to enrich the PTMs of interest for proteomic analysis. These strategies mainly include affinity-based chromatography methods that fall under the category of chemical proteomics. They are mainly based on the covalent capture of the PTM moiety by selective chemical probes. Specific affinity matrices are available to capture different types of PTMs. For instance, we can mention metal ions such as Fe^3+^, Ti^4+^, or Zr^4+^ on carrier resins for phosphate group enrichment, lectins for the enrichment of glycoproteins/glycopeptides and ubiquitin enrichment with certain epitope tags in cells (e.g. 6xHis, HA) or a nickel column. However, affinity-based capture suffers from drawback of non-specific protein bindings. For this issue, chemical labeling strategy has emerged which relies on the specific covalent binding of PTMs to chemical probes (146). Large-scale mass spectrometry results are followed by computational data analysis and also database searching algorithms which enable protein and PTMs identification and quantitation. PTMs are identified by comparing the experimentally measured molecular masses of peptide sequences with the *de novo* calculated masses obtained from the amino acid sequence of the proteins. Specific mass shifts can define various post-translational modifications. For instance, a mass shift of +80 Da for phosphorylation, +80 Da for sulfation, -131 Da for proteolytic removal of methionine, +16 Da for oxidation of methionine, or -2 Da for a disulfide bond can be observed. Moreover, some modified amino acids exhibit specific diagnostic m/z signals in MS/MS spectra (147). With the explosive generation of data from PTM identification studies over the past decades, the development of PTM-related databases was inevitable. These databases cover various aspects of PTM-related data, such as databases containing MS/MS results of PTM identification and localization, PTM prediction, disease-related PTMs, PTM ontology and network analysis, literature mining for PTMs, and evolutionary information of PTMs (148). PhosphoSitePlus (https://www.phosphosite.org/) is a comprehensive database of information on post-translational modifications of proteins, especially phosphorylation and acetylation. PTMD (http://ptmd.biocuckoo.org/) is a database of manually curated disease-associated post-translational modifications. PHOSIDA (http://www.phosida.com) is a phosphoproteome database containing information on phosphosite structure, evolution, and prediction. It also allows data retrieval for other PTMs, including acetylation and N-glycosylation. O-GLYCBASE (http://www.cbs.dtu.dk/databases/OGLYCBASE/) is also a database for glycoproteins with O-linked glycosylation sites for the proteins with at least one experimentally verified O-glycosylation site according to SwissProt database. There are also other databases containing information on various types of PTMs, including phosphorylation, glycosylation, ubiquitination, acetylation, and more.

### 4.1 Histone modifications and gastrointestinal cancers

GCs, including stomach, liver, colorectal, esophageal, and pancreatic cancers, accounts for more than 25% of all cancers, with an increasing prevalence over the past decade (149). Smoking, alcohol consumption, unhealthy dietary habits and lifestyle, and environmental factors, in addition to genetic aberrations, are major risk factors for GCs (150). Over the past decades, major advances have been made to understand the role of epigenetic alterations, including DNA methylation, histone modifications, and non-coding RNAs in carcinogenesis. Moreover, DNA or histone modifications may serve as potential diagnostic, prognostic, or therapeutic biomarkers for a variety of cancers, including GCs. Inhibitors of HDACs or other modifying enzymes such as histone demethylases may serve as novel therapeutics for cancer (151). Comprehensive molecular characterization of cancers using genomic, transcriptomic, epigenomic, and proteomic approaches has also opened up a new avenue for molecular diagnosis of GCs (152). The epigenome pattern can also be used to explore the mechanisms involved in the regulation of gene expression and fundamental pathways underlying GC pathogenesis. In this section, we summarize the current knowledge on the role of histone modifications in GCs pathogenesis and as GCs biomarkers, as well as proteomic approaches that have greatly contributed to expanding our knowledge in this field.

### 4.2 Histone modifications in gastric cancer

Gastric cancer is the 5th most common cancer and the 4th leading cause of cancer-related deaths worldwide in 2020 (149). Gastric cancers are divided into two major subsets, histologically; while most gastric cancers arise in distal regions of the stomach, about 18% of all gastric cancers occur in the part of the stomach adjoining the esophageal-gastric junction (153). Each type of gastric cancer results from the aggregation of several genetic and epigenetic changes (154). A deeper understanding of epigenetic alterations such as histone modifications and their potential therapeutic role may be helpful in gastric cancer treatment. Among the risk factors for this cancer, Helicobacter pylori (Hp) is responsible for approximately 75% of gastric cancers (155). It has been found that Hp causes dysregulation of histone modifications in epithelial cells and macrophages in the stomach, leading to the development of gastric cancer. In this regard, Yang et al. revealed that infection with Hp induces the H3S10 phosphorylation and facilitates gastric tumor formation (156). HP infection is correlated with local production of cytokines, of which, IL-6 is overexpressed at the margin of gastric ulcer in H. pylori-positive gastritis. A study conducted by Pathak et al. showed that HP induces phosphorylation of H3S10 in macrophages, enhancing the expression of IL-6 through binding to the promoter of this gene, which finally leads to Hp-induced gastritis (157). Fehri et al. found that H. pylori reduced the phosphorylation levels of H3S10 and H3T3 to regulate cell (158). On the other hand, aberrant epigenetic modifications affect cell functions, especially in digestive system cancers (159). Most of the researches on the effect of histone modifications in the development of gastric cancer are related to methylation, acetylation, and phosphorylation. In this regard, Ono et al. studied the status of histone H4 acetylation in gastric cancer tissues and corresponding non-cancerous mucosa (160). They observed decreased levels of acetylated histone H4 in gastric carcinoma compared with non-neoplastic mucosa. These findings suggest that low levels of histone acetylation may be significantly correlated with the development and progression of gastric carcinoma, possibly via aberrant regulation of gene expression (160). Both DNA methylation and histone modifications are involved in the silencing of key tumor suppressor genes and the activation of cancer-related oncogenes (161). On the other hand, the relationship between acetylation and invasion, lymphatic metastasis, and tumor stage has been confirmed. One study showed that hypoacetylation in histone H3 in the *p*21*^WAF^* ^1/^ *^CIP^* ^1^ promoter was correlated with reduced *p*21*^WAF^* ^1/^ *^CIP^* ^1^ expression in gastric carcinoma (162). In addition, high expression of HDAC1/2 was also found in gastric carcinoma, although it was not associated with treatment response or overall survival (OS) (163). In patients who responded to treatment, high levels of HDAC1 expression was correlated with worse OS. Therefore, expression of HDAC1 and -2 are not helpful to predict response to treatment or survival in gastric cancer patients treated with neoadjuvant therapy, but HDAC1 expression may be beneficial for risk stratification in patients with disease that responds to therapy (163). It is also noteworthy that HDAC4 is upregulated in gastric cancer and is associated with a poor prognosis. Therefore, targeted therapy based on HDAC4 would represent a novel approach for gastric cancer management (164). The majority of investigations on role of histone methylation in gastric cancer development are markedly connected with methylation of H3 and H4. Liu et al. reported that the histone H3 methylation contributes to the development of gastric cancer through downregulation of NGX6 in cancerous cells (165). Evidence suggests that downregulation of NGX6 gene mediates the development and progression of gastric cancer. However, further investigations are required to evaluate the precise mechanism of *NGX6* in the development and progression of gastric cancer (165). Another study evaluated the variations of H3K27me3 in CpG island regions comparing gastric cancerous and matched non-cancerous tissues (166). The results showed that significant changes of H3K27me3 occurred in cancer tissues, which may help to better understand the molecular mechanisms involved in the pathogenesis of gastric cancer. The work of Park et al. showed that triple methylation of H3K9 was associated with cancer stage, lymphovascular invasion, recurrence, and OS rates (167). Matsukawa et al. found that EZH2 was overexpressed in gastric cancer and was associated with tumor size, tumor invasion, lymph node metastasis, and clinical stages. In addition, multivariate survival analysis offered that elevated levels of EZH2 correlated with poor prognosis (168). The role of histone phosphorylation in gastric cancer development is less studied, however, high levels of H3 phosphorylation have been detected in gastric cancer (169). Moreover, H3 phosphorylation is beneficial and necessary for the anticipation of prognosis in gastric cancer and is associated with vascular invasion, lymphatic metastasis, and histological types. Expression profiling of histone-modifying genes during gastric cancer progression revealed decreased expression of HDAC5 during gastric cancer progression (170). These findings suggest an important role for epigenetic-based therapy in cancer.

Profiling and characterizing of histone isoforms and their post-translational modifications by proteomics-based strategies may be beneficial to uncover the molecular and cellular mechanisms underlying gastric cancer development. Lysine lactylation (Kla) is a newly discovered histone PTM that plays a key role in regulating gene expression (127). Recently, Yang et al. applied a comprehensive analysis including LC-MS/MS to profile the lactylated proteins in gastric cancer AGS cells (171). They also identified multiple Kla sites on non-histone proteins. In addition, they detected 27 core histone Kla sites, including eight previously unknown sites. Interestingly, KEGG pathway analysis showed that these proteins were significantly enriched in spliceosomes. On the other hand, they also observed that Kla was more abundant in gastric tumors than in adjacent tissues, and high levels of Kla in gastric cancer samples were linked to the poor prognosis (171). These findings suggest that Kla may be a useful prognostic biomarker for gastric tumors. Song et al. conducted a large-scale proteomic analysis of 30 gastric cancers and 30 matched healthy tissues using a label-free global proteome profiling. Analysis of differentially expressed proteins by the ingenuity pathway analysis indicated that the sirtuin signaling pathway was the pathway significantly activated in gastric cancer tissues (172). Sirtuins are members of the class III histone deacetylases family (173). Sirtuins are involved in the generation of cancer cells, which are capable of self-renewal and differentiation, leading to tumor growth (174). The newly identified PTMs and their associated target proteins are potential biomarkers or possible therapeutic targets for gastric cancer in the future. However, further studies are needed to elucidate the role of these alterations in the development of gastric cancer.

### 4.3 Histone modifications in colorectal cancer

Colorectal cancer (CRC) is the third most common malignancy and the second cause of cancer-related death worldwide (175). Colorectal carcinogenesis is a complex multistep process affected by various factors including aberration in genetic and epigenetic regulation. is an important mechanism in the occurrence and progression of CRC (176). Abnormal levels of histone modifications such as acetylation, methylation, and phosphorylation at specific residues are involved in various types of cancers, including CRC. Moreover, inhibitors targeting histone-modifying enzymes represent a potential cancer therapy (177). There is evidence that histone modification changes can impair the regulation of gene expression and contribute to the formation of colorectal cancer. Abnormal histone acetylation has been implicated in the pathogenesis of CRC and may influence clinical outcomes (178). Ohshima et al. investigated the role of mitochondrial function in CRC cells and demonstrated that mitochondria can induce histone acetylation (179). Therefore, investigating the related molecular mechanisms is important for developing diagnostic and therapeutic strategies for CRC (176). A better understanding of the underlying mechanisms may help to develop histone-related biomarkers, targeting histone-modifying enzymes, and investigating anti-tumor HDAC (histone deacetylase) inhibitors. Karczmarski et al. applied LC-MS/MS technique to quantify global changes in histone PTMs for CRC samples (180). In this study, 96 modified peptides were identified by a bottom-up proteomic, with 41 distinct PTM sites among them. The intensity of modifications was determined for 33 sites, of which 4 were significant. The results of this study demonstrate for the first time that H3K27 acetylation is significantly increased in CRC samples. They used MS and Western blot analyses and identified that histone H3 lysine 27 acetylation (H3K27Ac) was upregulated in CRC tissue samples compared to normal tissues. Wang et al. used a proteomic approach using SILAC-labeled samples for LC–MS/MS analysis to investigate the crosstalk between lysine phosphorylation and the acetylome in HDAC inhibitor romidepsin-treated colon cancer cells (181). They identified lysine acetylation and phosphorylation sites and quantified target up/downregulation. Romidepsin, as a histone deacetylase inhibitor, exerts its antitumor effect in colon cancer cells through changes in protein modification (181). Shen et al. used quantitative acetylated proteomics to compare the expression levels of acetylated proteins between CRC primary tissues and CRC with liver metastases. They identified and quantified the acetylation sites of the associated proteins and revealed differentially expressed acetylated proteins involved in CRC with liver metastasis (182). In this study, the most significantly altered acetylated histones (HIST2H3AK19Ac and H2BLK121Ac) and acetylated non-histones were reported. Recently, Zhang et al., using a label-free quantitative proteomic approach, analyzed the SIRT2 (an NAD-dependent deacetylase) acetylome of human CRC HCT116 cells and revealed various SIRT2 substrates (183). Histone H3 is considered as a secondary hub in the protein interaction network of the acetylated proteins involved in transcription regulation, while histone H2B is considered as a secondary hub in the protein interaction network of the acetylated proteins involved in the DNA damage response. Milli et al. performed a quantitative proteomic analysis to study the effect of HDAC inhibitor RC307 in HCT116 CRC cells and identified modulated proteins (184). Serum proteome profiling of colorectal cancer patients helped to identify SETD7 as a potential serum biomarker (185). Histone lysine methyltransferase SETD7 catalyzes methyl transfer from S-adenosylmethionine to the lysine residues on histones. Dysregulation of this methylation process is an important epigenetic mechanism in the development of cancer (186). The LC-ESI/MS and western blot analysis by Fraga et al. showed loss of acetylation at H4-Lys16 and trimethylation at H4-Lys20 in human cancer cells, including colon cancer (187). Current studies have shown that proteomics is an important tool for the discovery of histone-related biomarkers, targeting histone-modifying enzymes, and studying anticancer HDAC inhibitors.

### 4.4 Histone modifications in liver cancer

Liver cancer ranks among the top five leading causes of cancer-related mortality worldwide. According to the American Cancer Society 2022 statistics, more than 40,000 new cases will be diagnosed and nearly 30,000 deaths from liver cancer will occur in the United States in 2023. In addition, more than 800,000 people are diagnosed with liver cancer each year worldwide. The most important risk factors for liver cancer development include smoking, alcohol consumption, food-borne toxins such as aflatoxin B1, metabolic disorders, chronic infection induced by hepatitis B or C virus (HBV/HCV), and trichloroethylene. Type 2 diabetes and obesity have also been observed to promote the occurrence and progression of liver cancer (188). Hepatocellular carcinoma (HCC) is one of the most common digestive system malignancies which occupies more than 90% of primary liver cancer cases, having very poor prognosis (189, 190). It is difficult to be examined in early stages and therefore, most of the HCC cases develop to an advanced stage after symptoms occur, due to its rapid progression and high metastasis. Genetic and epigenetic modifications are key regulators of genomic aberrations involved in tumorigenesis mechanisms of HCC. Histone modifications are extensively observed in the progression of HCC. Moreover, novel therapies are being developed for HCC based on epigenetic regulatory mechanisms that have great potential for the treatment of patients in the future. Gene and Protein modifying enzymes, such as DNA methyltransferases (DNMT) and histone deacetylases (HDACs), are key enzymes overexpressed during HCC progression and may therefore serve as important targets for the development of alternative anticancer therapies for HCC. HDAC inhibitors are being developed for the treatment of HCC, which reactivate the expression of tumor suppressor genes, induce apoptosis and differentiation, and also inhibit tumor growth, angiogenesis, and metastasis (151). HDAC inhibitors such as panobinostat and sodium butyrate have been shown to inhibit HCC tumor growth (191, 192). Panobinostat, SAHA analogues, valproate and droxinostat have been shown to be HDAC inhibitors that induce apoptosis in hepatocellular carcinoma (193). Panobinostat, SAHA and santacruzamate A also increase the sensitivity to chemotherapy, radiotherapy or targeted therapies (194). HCC development is divided into the early and late stages. Inflammatory responses such as cytokine secretion play an important role in the development of early-stage HCC. Chronic inflammatory responses such as cirrhosis and necrosis are also key players in the progression of the late stage HCC. Epigenetic regulations, including DNA methylation and histone modifications, have been shown to be involved in the development of HCC in both early and late stages (195). Histone H3 lysine modifications mainly acetylation and methylation modulate some transcription factors which target genes such as VASH2, fatty acid synthase, RIZ1, MPP1/3, FBP1, and YAP that play a role in the metabolism, angiogenesis, and metastasis of HCC patients. Alcohol exposure, which is a major risk factor for liver cancer, has been shown to alter histone methylation and acetylation patterns (196). In a study by Bardag et al., alcohol-treated rats showed increased levels of histone H3K18 and H3K9, reduced levels of some nucleus proteins such as phosphoc-Jun, pERK, p38, phospho-SAPK/JNK, and increased amounts of β-catenin. The increase in β-catenin in hepatocytes suggests that activation of the canonical WNT/β-catenin pathway may be involved in the formation of liver tumors. Simultaneous increased levels of histone acetyltransferase (HAT) p300 and the deacetylase, SIRT1, were also observed in the chronically alcohol-fed rats. Methylation in histones H3K4 and H3K27 was also observed in alcohol-fed animals (197). High exposure to Aflatoxins as another risk factor of HCC, can also alter the histone modifications, causing HCC. The mechanism of HCC induction due to aflatoxin exposure lies on the binding of aflatoxin to the DNA at CpG sites by means of MeCP2 protein, which recruits enzymes such as histone deacetylases (HDACs) that condense the chromatin, and thus leads to hindering of the gene transcription (198). Histone methyltransferase/demthylase enzymes, mainly lysine and arginine methylases/demethylases are shown to be closely related to HCC progression. It was found that high expression of arginine methyltransferase 9 is related to invasion and metastasis in HCC by inducing epithelial-mesenchymal transition and activating PI3K/AKT signaling pathway (199). Other studies also showed the role of arginine methyltransferase 5 and 1 in the progression of liver cancer (200, 201). Histone lysine methyltransferase 6A (also known as EZH2) plays an important role in the development of HCC (202). Histone lysine demethylase 1A (known as LSD1), which regulates the level of dimethylation in the promoter of H3K4, promotes the tumorigenicity and chemotherapy resistance in HCC (203). Histone-modifying genes/proteins could also be used as biomarkers of liver cancer. High throughput “omics” approaches, mainly transcriptomics and proteomics, have attracted great attention in this regard. In a proteomic study by Wang et al., the acetylated and methylated histone H3 profile were studied in different human HCC cell lines HepG2, 97H, LM3, and 97L, compared with L02 normal liver cells (204). Histones were enriched from the cells by acid extraction, followed by separation on a C18 reversed-phase chromatographic column, and determination by an LTQ-Orbitrap mass spectrometer. The collected H3 histones were then identified by MALDI-TOF-MS. Modified peptides with expression fold change>2 were defined as differentially expressed. The results showed quantitative information on 6 acetylated and 4 methylated peptides in three cell group samples ([HepG2 and L02], [97H and HepG2], and [LM3 and 97L)]. The histone H3 significantly altered acetylated peptides included K9STGGK14APR, KQLATK23AAR, K18QLATK23AAR, and K27SAPSTGGVK, among which the most significant methylated peptides included K9STGGK14APR, K27SAPATGGVK, and EIAQDFK79TDLR. Another proteomic study was conducted by Hu et al. who studied the acetylome profile of solid tumors and adjacent noncancerous tissues from eight adult patients diagnosed with primary hepatocellular carcinoma (205). Protein lysates were digested with trypsin and enriched by immunoaffinity techniques. Mass data acquisition was performed on an EASY-nLC 1000 system connected to an Orbitrap Fusion mass spectrometer equipped with an online nano-electrospray ion source. They identified 2,060 acetyl sites in 733 proteins using the acetylomic approach. The results showed a compartment-specific expression of acetylated histones according to cellular components Gene Ontology term. It was observed that hyper-acetylated proteins were preferentially enriched in the mitochondrial metabolic pathways. In a proteomic study by chai et al., a quantitative acetylome analysis revealed several histone modifications that may predict survival-associated prognosis in hepatitis B-related hepatocellular carcinoma (206). Acetylated peptides from normal, paracancerous, and HCC liver tissues were enriched by immunoaffinity precipitation followed by identification by an online nanoAcquity ultraperformance liquid chromatography coupled with an Orbitrap Fusion Tribrid mass spectrometer. They identified 6781 acetylation sites of 2582 proteins, of which, 15 proteins were multi-acetylated with more than 10 lysine residues. Moreover, H2BK120ac, H4K77ac, and H3.3K18ac were significantly associated with poor survival and higher recurrence in an independent cohort of HCC patients. A proteomic study was performed by Xu et al., in which the lysine acetylome from HCC tissue was isolated by immunoprecipitation and characterized using a timsTOF pro mass spectrometer (207). They identified 1003 lysine-acetylation sites with differential acetylation levels between HCC and normal adjacent tissues. Liu et al. performed a comparative proteomic analysis to profile protein methylome in 5-fluorouracil resistant hepatocellular carcinoma (208). Peptide fractionation was performed using a solid phase extraction (SPE)-SCX followed by a nano LC–MS/MS for both resistant and sensitive HCC cell line Bel. They reported that five methylation types, including three lysine (Kme1, Kme2, and Kme3) and two arginine (MMA, DMA) methylations were significantly altered between 5-Fu sensitive and resistant cells. A proteomic study by Zhao et al. revealed the profile of more than 300 significantly expressed lysine acetylome in hepatocellular carcinoma tissues compared to normal liver tissues by affinity enrichment coupled to LC-MS/MS analysis (209). Most of the altered acetylated proteins included mitochondrial and cytoplasm non-histone proteins, mainly involved in oxidative stress, metabolism, and signal transduction processes. MALDI Imaging Mass Spectrometry (MALDI IMS) is another proteomic approach which was utilized by Pote et al. to reveal the histone H4 modifications related to microvascular invasion in hepatocellular carcinoma (210). Among the 28 significantly altered modifications, N-term acetylated histone H4 dimethylated at K20 and also both dimethylated at K20 and acetylated at K16 were confirmed by immunohistochemistry and western blot analysis.

### 4.5 Histone modifications in pancreatic cancer

Despite the rapid growing of medical technologies, pancreatic cancer (PC) still remains a highly lethal GCs (211). Several PTMs involved in the epigenetic regulation of pancreatic cancer are being investigated as potential targets for developing diagnostic and therapeutic strategies. Lu et al. applied the liquid chromatography-tandem mass spectrometry (LC-MS/MS) technique to identify lysine 2-hydroxyisobutyrylation (K_hib_) sites (as a novel PTM) on modified proteins in pancreatic cancer (212). The results showed a total of 27 K_hib_-modified sites in histones and provided the first profile of the lysine 2-hydroxyisobutyrylome in PC. Bioinformatics analysis showed that K_hib_ protein was mainly enriched in metabolic pathways. The results suggest that K_hib_ modification may affect PC metabolism and contribute to cancer development. Bauden et al. used LC-MS/MS to obtain a profile of histone-related PTMs in pancreatic ductal adenocarcinoma (PDAC). They suggested H1.3 expression as an epigenetic biomarker for PDAC (213). Histone acetylation and deacetylation is a process regulated by two families of enzymes, histone acetyltransferase (HAT) and histone deacetylase (HDAC). The balance between acetylation and deacetylation is important and can control various mechanisms of health and disease. Zhang et al. studied the molecular changes of HAT and HDAC genes in GCs, including pancreatic cancer. They extracted the protein interactions between HAT and HDAC genes and constructed a protein-protein interaction network. The resulting network was found to be closely correlated with certain pathways involved in pancreatic cancer (214). Cecconi et al. also applied a proteomic approach to study the synergistic effects of the histone deacetylase inhibitor trichostatin A (TSA) and the DNA methyltransferase inhibitor, 5-aza-2’-deoxycytidine (DAC), in pancreatic cancer (215). They showed that the combined treatment of pancreatic tumor cells with TSA and DAC exerted a strong synergistic inhibitory effect on pancreatic tumor cells proliferation. Their proteomic analysis also demonstrated that the most important targets and pathways involved in the pancreatic cancer growth inhibition included mitochondrial apoptotic pathway, proteasome, caspase-related proteins, p53, and Ras-related proteins.

## 5 Concluding remarks, challenges, and future directions

Histone modifications are one of the epigenetic changes that have attracted major attention due to their influence on cancer development and treatment. Histone PTMs and DNA methylation not only play important roles in the development of various diseases but also play a key role in regulating cellular processes such as gene transcription, cell replication, DNA repair, and DNA repair. Therefore, the study of histone modifications is a new and growing field that aims to improve our understanding of the key processes of carcinogenesis and tumor progression. The identification of dysregulated histone modifications may provide a golden opportunity for the development of new therapeutic and preventive strategies against cancer. Despite extensive research, GCs remains a major health problem and remains the leading cause of cancer morbidity and mortality. Over the past decades, great progress has been made in understanding the role of epigenetic changes in cancer, including GCs. In this regard, profiling of PTMs and target genes associated with PTMs is expected to help understand the pathogenesis and develop new therapies for GCs. High-throughput proteomics is a golden approach that plays a central role in identifying and quantifying post-translational modifications. Mass spectrometry-based methods are proving to be the most effective methods for identifying PTMs to date. Several recent studies have evaluated the potential role of histone modifications in GCs, mainly through proteomic approaches. Changes in histone modifications may be used as novel biomarkers for predicting cancer metastasis and targeted therapies. However, further studies are needed to verify the correlation between early epigenetic changes and clinical features that may provide pathways for achieving better clinical outcomes. There are several challenges to elucidating the role and mechanisms of HMs in cancer development and progression. For example, histone modifications are dynamic events. In addition, HM catalyzing enzymes may also catalyze some modifications on non-histone proteins that influence p53, Rb, and etc. Another important challenge is that crosstalk among HMs can lead to independent, competitive, or synergistic effects. On the other hand, mass spectrometry-based approaches typically present data on the overall level of histone modifications, but do not localize modifications in specific regions of the genome. Meanwhile, integrating MS-based approaches with ChIP-seq analysis of histone modifications will provide a more comprehensive view of the different types of epigenetic dysregulation associated with cancer. Finally, combining data obtained through different omics-based approaches, including proteomics and genomics as well as transcriptomics, will be essential to fully decipher the results obtained from these powerful approaches and elucidate how epigenetic aberrations may influence cancer phenotypes.
